# Commentary: Reduction in C-Peptide Levels and Influence on Pharmacokinetics and Pharmacodynamics of Insulin Preparations: How to Conduct a High-Quality Euglycemic Clamp Study

**DOI:** 10.3389/fphar.2022.843658

**Published:** 2022-02-18

**Authors:** Hui Liu, Hongling Yu, Ting Li, Yerong Yu

**Affiliations:** ^1^ Department of General Practice, West China Hospital of Sichuan University, Chengdu, China; ^2^ Department of Endocrinology and Metabolism, West China Hospital of Sichuan University, Chengdu, China

**Keywords:** euglycemic clamp, pharmacokinetics, pharmacodynamics, C-peptide, insulin preparations

In a recent issue of this journal, Yi Tao and coworkers published a comprehensive article (Tao et al., 2021) focusing on the effect of C-peptide in a euglycemic clamp on the evaluation of the pharmacokinetics (PK) and pharmacodynamics (PD) of insulin preparations, therefore elucidating the method to perform a high-quality euglycemic clamp.

We completely agree that suppression of endogenous insulin is of paramount importance in euglycemic clamp in healthy volunteers (European Medicines Agency, 2015; Heise et al., 2016). The topic of evaluating the reduction of postdose C-peptide and its influence on the PK/PD of the study preparations is interesting and deserves research. The work by Tao et al. (2021) included a total of 39 euglycemic clamps lasting for up to 24 h evaluating the PK/PD of insulin glargine in healthy volunteers. These volunteers were divided into three groups according to the reduction of C-peptide levels: group A (ratio of C-peptide reduction <30%, *n* = 13), group B (ratio of C-peptide reduction between ≥30% and <50%, *n* = 15), and group C (ratio of C-peptide reduction ≥50%, *n* = 11). The PK/PD of insulin glargine and the clamp quality index were compared among the three groups.

This article is of significance considering improving the quality of the clamp study. In this letter, based on the reported studies and our experiences (Heise et al., 2016; Li et al., 2012; Liu et al., 2021; Li et al., 2021), we intend to provide different opinions on the method and some results of this article. First, the EMA guideline recommends using specific assays that are capable of distinguishing between exogenous and endogenous insulin to determine the concentration of insulin analogs (European Medicines Agency, 2015). In the published work by Yi Tao and colleagues, the insulin glargine level was determined by a validated liquid chromatography–tandem mass spectrometry (LC-MS) method which is known for its specificity and has been used in the bioanalysis of peptides, including insulin and insulin analogs (Xu et al., 2017; Xu et al., 2014; Thomas et al., 2014). In the previous report, Xu et al. (2014) conducted selectivity experiments for the LC-MS/MS assay, demonstrating no cross-talk among glargine, M1, M2, bovine insulin, and human insulin at the 50 ng/ml level of each analyte. No interference was detected from multiple lots of human plasma, including those from diabetic patients. In addition, spiking 500 μU/ml human insulin did not have any impact on the assay results. As a result, in the analytical method applied to determine insulin glargine level in the work by Yi Tao and coworkers, insulin glargine does not cross-react with endogenous insulin. Second, in the absence of specific assays for insulin preparations evaluated by euglycemic clamps employing healthy volunteers, C-peptide should be measured in parallel to insulin concentrations throughout the experiment. Total insulin concentration should be detected in other nonspecific analytical methods, e.g., a radioimmunoassay method (Zhang et al., 2017; Linnebjerg et al., 2015), and minus the endogenous insulin using the ratio of C-peptide to endogenous insulin by Owen’s method (Owens, 1986) to roughly calculate the exogenously injected insulin. Under these conditions, the different extent of C-peptide inhibition may affect endogenous insulin-corrected PK data, but it still needs to be proved. Third, GIR is recognized as a surrogate for PD in clamp studies. Total glucose infusion quantity is influenced by many factors (e.g., dose of insulin glargine, insulin sensitivity, “clamped” glucose level, absorption kinetics of insulin glargine, and the influence of endogenous insulin). It is no doubt that achieving a higher “clamped” glucose level requires infusing more dextrose. In this published clamp study, the average “clamped” glucose levels were 5.2, 5.1, and 4.8 mmol/L when the target glucose levels were 4.98, 4.99, and 4.90 mmol/L in groups A, B, and C, respectively. The average “clamped” glucose levels were 0.3–0.4 mmol/L higher in groups A and B than those in group C. Not only insufficiently suppressed endogenous insulin but also higher “clamped” glucose levels will likely trigger additional glucose infusion requirements to maintain the blood glucose. Direct comparison of the main PD parameter, the area under the curve of GIR from 0 to 24 h (AUC_GIR,0–24 h_), without adjustment for other confounding factors invalidates the results. Finally, the study published by Yi Tao and colleagues only included a relatively small sample size (total *N* = 39), resulting in much smaller samples in subgroups. Previous reports (Heise et al., 2012; Heise et al., 2018) demonstrated that insulin glargine (100 U/ml) showed a higher pharmacodynamic variability, which might indicate a higher variability in pharmacokinetics. The statistical power may not be strong enough to validate the interpretation of the results due to the small sample size. The limited sample size in subgroups raises caution with the interpretation of the results.

Long-acting insulin analogs such as insulin glargine will consistently remain at a relatively low level for a rather long period; therefore, interference of endogenous insulin makes sense in clamp studies conducted in patients with type 2 diabetes or the healthy people. C-peptide will not influence any PK parameters when the bioanalytical method of insulin preparation is performed with high specificity and precision, and free from the interference of endogenous insulin. Aiming to investigate the effect of the extent of C-peptide inhibition on the PK/PD of exogenous insulin preparations, PK data may be obtained from the C-peptide correction method. PK/PD data should also be obtained from a respectable sample size to reflect the real performance of the treatment. Further work is indeed needed.
